# Influence of Urban-Growth Pattern on Air Quality in China: A Study of 338 Cities

**DOI:** 10.3390/ijerph15091805

**Published:** 2018-08-22

**Authors:** Yanchuan Mou, Yan Song, Qing Xu, Qingsong He, Ang Hu

**Affiliations:** 1 College of Architecture and Environment, Sichuan University, No. 29 Jiuyanqiao Wangjiang Rd, Chengdu 610064, China; yanchuan2015@outlook.com (Y.M.); suzie.xuqing@gmail.com (Q.X.); 2 Department of City and Regional Planning, University of North Carolina at Chapel Hill, Chapel Hill, NC 27599, USA; ys@email.unc.edu; 3 College of Public Administration, Huazhong University of Science & Technology, 1037 Luoyu Road, Wuhan 430074, China

**Keywords:** urban growth pattern, urban form, air quality, spatial regression, Chinese cities

## Abstract

Air pollution in China is a serious problem and an inevitable threat to human health. This study evaluated the relationship between air quality and urban growth pattern in China by conducting empirical research involving 338 prefecture-level and above cities. Spatial regression techniques considering spatial autocorrelation were applied to correct the calculation bias. To obtain local and accurate results, a conception of eight economic zones was adopted to delineate cities into different groups and to estimate regression separately. An additional six urban form and socioeconomic indicators served as controlling variables. Significant and positive relationships between the aggregated urban growth pattern index and air pollution were observed in Northeast China, northern coastal China, and Northwest China, indicating that a high degree of urban aggregation is associated with poor air quality. However, a negative parameter was obtained in southern coastal China, showing an opposite association on urban aggregation and air quality. Nonsignificant connections among the other four zones were found. The findings also highlighted that land use mix, population density, and city size exerted varied and significant influence on air quality across eight economic zones. Overall, this study indicated that understanding the quantitative relationships between urban forms and air quality can provide policymakers with alternative ways to improve air quality in rapidly developing China.

## 1. Introduction

**Figure 1:**
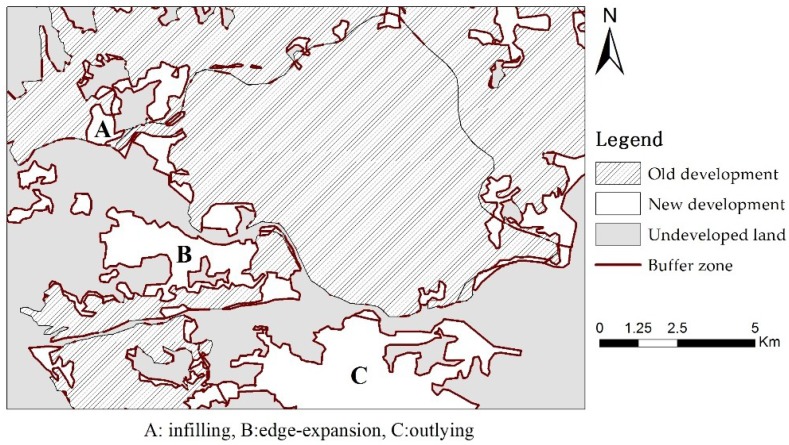
Three types of urban growth pattern (part of Jinan City).

With the implementation of the Reform and Opening-up policy, China has experienced rapid urbanization with an increase from 20.16% to 54.77% between 1981 and 2014 [1]. China is also experiencing a period of unprecedented urban expansion. The urban land area in China totaled 6720 ${\mathrm{km}}^{2}$ in 1981, and it increased to 49,900 ${\mathrm{km}}^{2}$ in 2014, representing an increase of 644% and highlighting a significant change, in accordance with the data released by the National Bureau of Statistics of China. The tremendous nationwide expansion of urbanized area resulted in various configuration types of urban development, and considerable attention has been paid to the research on dynamic development of urban morphology from the perspective of landscape ecology in recent years [2]. As an important index of landscape ecology, spatial patterns of urban growth refer to the locations of newly grown patches in developed lands. This indicator provides a powerful tool for understanding the evolutionary process of urban areas, identifying the degree of urban sprawl, and predicting urban growth, and has attracted considerable attention [3]. In general, urban growth involves three different spatial patterns, edge expansion, outlying, and infilling (Figure 1A–C). Specifically, edge expansion refers to the phenomenon of homocentric outspread, indicating a spatially subsequent expansion and extension of urban built-up areas. Outlying is characterized by the new urban lands occurring beyond developed areas. Infilling is introduced as developing the vacant land between established patches [4,5,6].

In general, the three urban growth patterns result in various effects on land spatial distributions, land use/cover change, efficiency of land use, and travel choices. Edge expansion causes redundancy in the low-level construction of urban infrastructure by dispersing investment opportunities, indicating a large occupation of farmland and low efficiency in land use [7]. For example, in Beijing’s rapid urban expansion process, significant urban growth has been observed on the fringe, especially in low-density gated communities and industrial development. As a result, the need for long-distance travel to external areas and the use of private vehicles on the city fringe have increased. In addition, edge expansion overburdens urban core areas, resulting in various urban disease types, such as environmental deterioration, traffic congestion, and housing shortage. Outlying invades prime agricultural and resource lands in the process, and leads to fragmented and piecemeal land development patterns. Several issues, including low-density residential development; insufficient land use; rigid separation of shops, homes, and workplaces; poor access from one place to another; and a heavy reliance on auto-mobiles, arise from outlying expansion [8]. Infilling is characterized by the following description: relatively high-density development close to or within the city core and aggregation of various workplaces and public/private services [9,10]. Infilling is an urban form that encourages walking and riding; it features low-energy consumption based on an efficiently developed public transportation system and a high degree of mixed land use [11,12]. As a result, infilling is incorporated into important urban planning and design concepts (smart growth and urban regeneration), whereas edge expansion and outlying are regarded as the main manifestations of urban sprawl.

Urban air pollution has rapidly emerged as a main environmental issue in China in recent years [13]. Data show that only three of the 74 key cities (4%) reached Class II of the Chinese National Ambient Air Quality Standard [14]. Simultaneously, air pollution has caused serious public health effects and economic damage in China [13]. In China, the cost of the health effects of air pollution approximated USD 1.4 trillion in 2010 [15]. Traffic exhaust has long been known to be one of the largest contributors to poor urban air quality for pollutants, such as PM_2.5_, carbon monoxide (CO), nitrogen oxide (${\mathrm{NO}}_{X}$), benzene, and ozone ($O_{3}$) [16,17]. This phenomenon is particularly true in China, where car ownership escalated to 488% in the last 10 years, and has reached to 290 million based on the 2016 *Yearbook of China Transportation & Communications*. As estimated, 24%, 20%, and 29% of the overall ${\mathrm{NO}}_{X}$, CO, and volatile organic compounds were contributed by vehicles in China at the country level, respectively, and they increased to approximately 40–70% at the urban level [18]. In addition, an urban forest can improve regional air quality by removing atmospheric pollutants, lowering air temperatures, and reducing building energy use and the consequent power plant emissions [19]. Previous research demonstrated that the total annual air pollution removal ($O_{3},{\mathrm{PM}}_{10},\;{\mathrm{NO}}_{2},\;{\mathrm{SO}}_{2},\;\mathrm{and}\;\mathrm{CO}$) by urban trees in the United States is estimated at 711,000 metric tons [19]. Therefore, the following hypothesis can be concluded from the context: urban growth patterns, as indicators related to travel choices and land use/cover change, are significantly associated with air quality.

As previously discussed, in comparison with edge expansion and outlying, infilling benefits air quality through two dominant paths—less private car dependency and improved open space preservation. However, with the expansion of the population amount and vehicle ownership, high-density development may increase the traffic volume and lead to heavy traffic congestion, which results in serious urban air pollution [20]. Of all of the countries in the world, China’s cities are characterized by high population agglomeration, specifically in megacities with a population density of up to more than 15,000/${\mathrm{km}}^{2}$ [21]. Therefore, the relationships between urban growth pattern and air quality in Chinese cities are indirect, and empirical studies are needed to reveal ambiguous association. Furthermore, the urbanization rate of China totaled 57.35% in 2016 and is predicted to reach 70.12% in 2030 [22]. Hence, in the next 15 years, urban areas will continually expand to accommodate a high number of rural–urban migration, leaving considerable space to shape their form. Therefore, an empirical analysis regarding the relationship between urban growth pattern and air quality is particularly needed and bears significance to rapidly developing China.

To test the relationship, this study analyzed the urban built-up area of 338 Chinese prefecture-level and above cities from 2005 and 2015 based on satellite imagery, and identified the aggregated index for all of the newly created patches within each city during this period. Six other widely used urban form and socioeconomic variables were used as controlling indicators. The remainder of this paper is organized as follows. Section 2 highlights the gaps in the indicators and methodologies used in the existing studies. Section 3 describes the variables and study area, and explains the research methodology in detail. Section 4 subsequently presents and discusses the regression results. Section 5 draws conclusions from the findings.

## 2. Literature Review

An increasing number of studies on the association between air quality and urban form have been conducted [23,24,25,26]. Table 1 summarizes the most widely used urban form indicators.

From a landscape perspective, landscape pattern refers to the spatial distribution and the combination of patches with differing sizes, shapes, and contents. Landscape process reveals the continuous and discontinuous changes in landscape patterns at the time–space scales. Landscape pattern and process are intrinsically related concepts and are keys to the theory and practice of landscape ecology [27]. Table 1 shows that all of the used landscape indices can only quantitatively reflect the landscape patterns for one single time point, lacking reflection on the dynamic process of land cover change. Urban growth pattern is an indicator that links patterns and processes and provides efficient information about urban development. It has thus has attracted lots of attention in recent years. For example, He et al. [28] explored the relationships between urban growth patterns and urban vitality. The results demonstrated that different urban growth patterns are associated with various kinds of urban vitality, indicating that cities may utilize these different urban expansion types to achieve targeted goals. As for this article, the use of an urban growth pattern index will provide a deep understanding about the evolution of urban morphology and its impact on air quality.

Most of the previous empirical studies on urban form and air quality have focused on cities in developed countries, and to our knowledge, existing analyses of Chinese cities are limited. More specifically, in the research of 157 Chinese cities, urban form is measured by six spatial metrics. The results showed that high population density and low urban continuity are commonly associated with good air quality through linear regressions [14]. The study conducted a comparatively comprehensive series of urban form metrics, and empirically demonstrated that urban form influences air quality in major Chinese cities. Liu et al. [31] explored the effects of urban form, measured by the compactness and elongation ratios on urban smog for 30 Chinese cities, through the use of a panel data analysis. The results indicated a significantly positive correlation when controlling for other independent variables. The studies, however, were based on global regression models and ignored spatial autocorrelation, which is derived from Tobler’s first law of geography, which states that “everything is related to everything else, but near things are more related than distant things” [32]. Urban air pollution is a function of economic activity within the city and is also a function of pollution of nearby cities, whose emissions are imported as a result of wind patterns [33]. For example, winds contribute 30% to 40% of Beijing’s air pollution by carrying pollutants from adjacent industrialized regions [34]. Autocorrelation in urban air quality data has been widely demonstrated and, if ignored, can lead to biased or misleading results [30]. Lu et al. [35] analyzed the relationship between the urban form and air quality of 287 Chinese cities on the basis of a geographically weighted regression model, which considers the geographical location in the intercepts and coordinates in the parameter estimates. With the rapid expansion of vehicle ownership, traffic exhaust has become the major cause of urban air pollution in the most recent years. Air pollution spreads from industrial and resource-based cities, such as Hebei and Shanxi, and has now become a critical issue across the whole country. For example, on the basis of the annual reports from the Chinese Ministry of Ecology and Environment, 60.5% of the prefecture-level cities reached Class II air quality standards in 2007, but the number decreased to 21.6% in 2015, highlighting the nationwide deterioration of air quality in recent years. Therefore, the data (2007) used in the article are not representative of the present relationship in such a rapidly developing country. In this context, spatial econometric models and the latest data are used to correct the autocorrelation bias and obtain accurate results.

## 3. Materials and Methods

### 3.1. Variables

#### 3.1.1. Air Quality Index (AQI)

Different indices have been used by a number of studies for air quality assessment [23,24,28]. AQI, as a simple and summary metric, is widely used by local authorities to provide information about local air quality and associated health advice for the public, and is also widely utilized in academic studies to measure the overall air quality [24,30,36]. From 2012 onward, more than 1400 monitoring stations were set to record hourly air pollution data across Mainland China. With the recorded data, the daily AQI for each city is calculated by considering six major air pollutants, which are regarded as key urban atmospheric pollutants (i.e., PM_2.5_, PM_10_, ${\mathrm{NO}}_{2}$, CO, ${\mathrm{SO}}_{2}$, and $O_{3}$). The AQI value runs from 0 to 500 and is divided into six categories as provided in Table 2.

Table 2 shows that a higher AQI value indicates a higher level of air pollution and more serious influence on human health. An AQI exceeding 100 indicates poor air quality from a public health perspective [30]. Daily AQI data are available on the website http://datacenter.mep.gov.cn/index. A total of 132,495 records for 338 cities in 2015 were collected using crawling techniques. Then, we counted the number of days where AQI > 100, and considered the value as indicator to measure the air quality. A high number of exceedance days in a city is associated with poor air quality.

#### 3.1.2. Aggregated Urban Growth Pattern Index (AUGPI)

The land use data for 2005–2015 were obtained from the National Land Use/Cover Database of China at the 1:100,000 scale. In accordance with the land resource and utilization attributes, six classes of land uses—cropland, woodland, grassland, water body, built-up land, and unused land—were identified [6]. A 30×30 m gridded database of land use classification, which is considered to be an accurate and reliable dataset for the monitoring, forecasting, and driving analysis of land use change at a regional scale, has been accomplished [37].

The growth pattern of newly created urban patches can be measured by identifying the common boundary between new and existing urban areas, or by exploring the composition of a buffer zone. On the basis of the theory, the landscape expansion index (LEI) was used by Liu et al. to analyze urban expansion quantitatively [38]. LEI divides the urban growth pattern into three types, namely, edge expansion, outlying, and infilling, and is widely recognized as an efficient tool to reveal the relationships between the spatial distribution of urban landscape and its evolution [39,40]. Therefore, we utilize LEI to identify urban growth pattern in 2005–2015 for 338 cities. LEI is calculated by Equation (1), as follows:

(1) $$\mathrm{LEI}=100\times \frac{A_{0}\;}{A_{0}+A_{V}}$$

where LEI is the landscape expansion index of a newly created patch, $A_{0}$ denotes the intersecting area of buffer zone and existing urban patches, and $A_{V}$ represents the intersecting area of buffer zone and non-developed land. In accordance with the buffer set criterion, that a small value is effective, a buffer size of 1 m was used in this study [38]. By definition, the LEI changes from 0 to 100. Urban growth pattern is defined as (1) outlying when LEI = 0, (2) edge expansion when 0 < LEI ≤ 50, and (3) infilling when LEI > 50. In general, more than one new patch has been created in a city. The total number of newly created patches in 2005–2015 reached 122,498. Therefore, the aggregated index over all of the patches should be evaluated for each city. The mean expansion index and area-weighted mean expansion index are introduced [38]. The latter was used in this article to calculate the AUGPI for a city on the basis of Equation (2), as follows:

(2) $$\mathrm{AUGPI}=\overset{N}{\underset{i=1\;}{\sum }}{\mathrm{LEI}}_{i}\times \frac{a_{i}}{A}$$

where ${\mathrm{LEI}}_{i}$ and $a_{i}$ represent the LEI and area of patch i, respectively, i⊂(1,N), N denotes the number of newly created patches, and A refers to the sum area of all of the new patches. AUGPI is a comprehensive and quantitative description of urban expansion, and a small value indicates less degrees of urban aggregation.

#### 3.1.3. Control Variables

On the basis of the findings from the literature review, the configuration of urban development has been demonstrated to be associated with air quality in China. To control for other urban form variables, four widely used indicators were selected in multiple perspectives, namely, land use mix, urban shape compactness, population density, and street connectivity.

Specifically, land use mix aims to quantify the heterogeneity of land uses in geographically defined areas, and a high mixed value decreases the long-distance travel demand for residents by offering additional nonresidential destinations nearby, which in turn reduces car usage [41]. Entropy is used to calculate the mixed index (*M*) on the basis of more than 23 million points of interest (POIs) from Baidu Maps, through Equation (3). The initial 12 POI types were aggregated into four general categories, residence communities, living and employment sites, transport facilities, and recreation sites, as follows:

(3) $$M=-\underset{i=1\;}{\sum }p_{i}\ln p_{i}\;\left(i=1,\;2,\;3,\;4\right)$$

where $p_{i}$ is the proportion of POI type i among all of the POIs. A high *M* value indicates additional POI types and a large land use mix of the patch. The area-weighted mean method was then applied to calculate the global *M* value of a city, by considering the weight of the area for all of the patches within the urban built-up areas.

Urban shape compactness is a key indicator to reflect urban structures from a land use perspective. A compact city is often considered to feature environmental and energy advantages through a concentrated urban development form [42]. The compactness index (*C*) is calculated in Equation (4), where P denotes the perimeter of the urban built-up area, and A represents the urban built-up area. A higher *C* value indicates a more compact city. Conversely, a low *C* value indicates a significant spatial dispersed urban layout, as follows:

(4) $$C=2\sqrt{\pi A\;}/P$$

Population density is generally recognized as one of the basic components of urban sprawl, whereas low-density development is associated with long vehicle miles traveled, which in turn results in a significant magnitude of vehicle emission and poor regional air quality [23,43]. Population density is measured by the number of persons per square kilometer within urban built-up areas.

Street connectivity is a measurement of the street density and is based on the ratio of the road surface area to the urban construction land area. In general, a higher ratio indicates better the connectivity and traffic capacity of a city.

**Figure 2:**
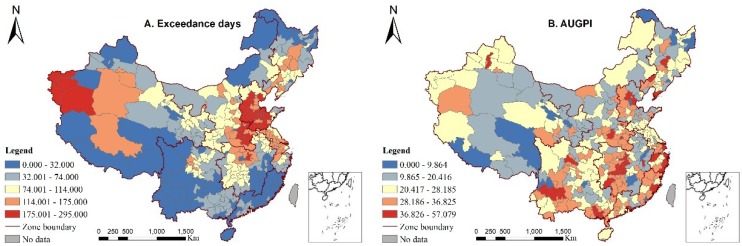

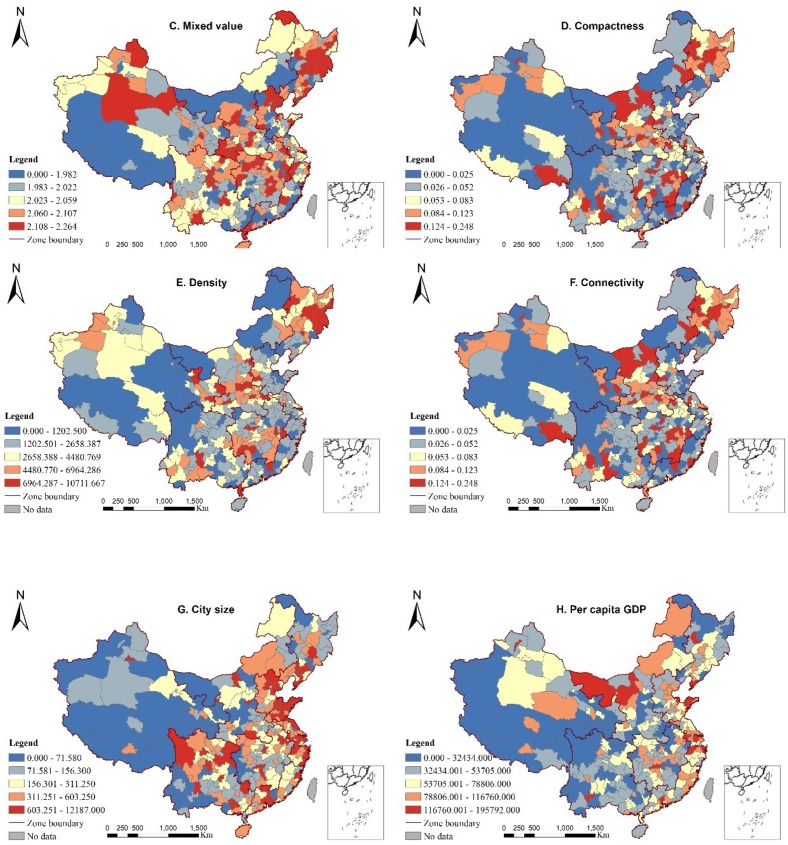
Spatial patterns of the variables across China.

Additional explanatory variables were included in this study, namely city size and per capita GDP. City size reveals the urban expansion speed, and a high value indicates that the expansion of urban areas for living and infrastructure have occupied additional green lands. Per capita GDP is a measurement of the city economic development. The data on population density, road surface area, and per capita GDP were obtained from the *China Urban Construction Statistical Yearbook*. Table 3 includes a summary of variable descriptions and data sources, and Table 4 and Figure 2 list the descriptive statistics and spatial patterns about the variables for the 338 cities (Detailed information about study area is introduced in Section 3.2).

### 3.2. Study Area

**Figure 3:**
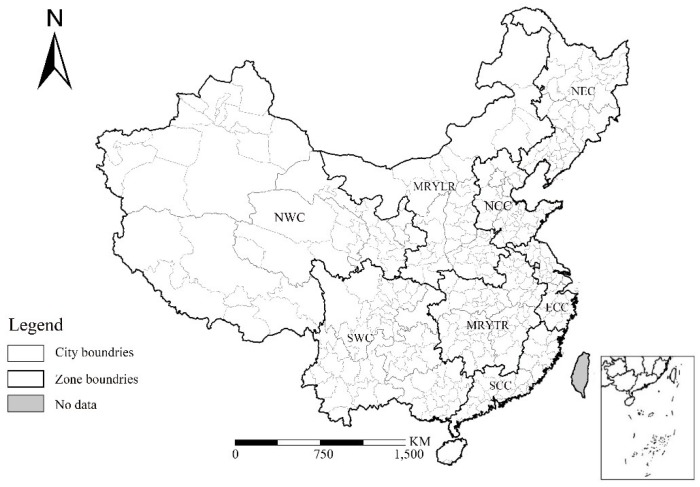
Administrative boundaries of the study area.

This research covers 334 prefecture-level cities (di ji shi) and four municipalities (zhi xia shi), with a total of 338 Chinese cities. A general definition of the Chinese administrative system is provided (i.e., prefecture-level cities rank below a province and above a county, and a municipality features the same political, economic, and jurisdictional rights as a province) [44]. The city boundary spatial data were derived from China’s second national land use survey. Considering that air quality is affected by nearby pollution sources because of wind patterns, local and accurate results will be obtained by delineating cities into different groups and estimating regression separately. This study adopted the conception of eight economic zones, which was proposed during the “11th Five-Year” period (2006–2010), and was classified on the basis of key characteristics, such as economic development, industrial structure, transportation system, and administrative division. The zones consist of Northeast China (NEC), northern coastal China (NCC), southern coastal China (SCC), eastern coastal China (ECC), the middle reaches of the Yellow River (MRYLR), the middle reaches of the Yangtze River (MRYTR), Southwest China (SWC), and Northwest China (NWC) (Figure 3). A significant difference in air quality was observed among the eight economic zones. For example, the mean exceedance days of NCC is 179.71, which is nearly eight times that of SCC. Table 5 shows the descriptive statistics for all of the zones. On the basis of these statistics, we hold the view that the relationships between air quality and independent variables in different zones relatively differ, and we should separately discuss the association.

### 3.3. Model Building

Moran’s I was applied to test the autocorrelation in the urban air quality data. The results show that Moran’s index is statistically significant with a 0.649 value. Furthermore, the regression residuals for non-spatial regression (in this context, we used ordinary least squares [OLS] model) were tested, and Moran’s I equaled 0.613 (*p*-value = 0.000). The results indicate the presence of spatial autocorrelation in the air quality geographic data. The model estimation error will be induced if spatial autocorrelation is ignored. Therefore, the interaction between cities should be considered in the model in order to correct the calculation bias. In general, the spatial weight matrix is applied to modify the basic linear model, and the following two ways are widely used to add spatial autocorrelation into the regression model:

(1) Spatial lag model (SLM)—The autocorrelation effect is attributed to the correlation between the dependent variable and its adjacency value, which is similar to the autoregressive form in the time series model. The lag term, ρWy, is added in the equation to eliminate the correlation. The model form is as follows:

(5) y=ρWy+xβ+ε

where y refers to the explained variable (urban air quality), x denotes the seven explanatory variables (AUGPI and controlling variables); W represents an n × n spatial weight matrix, where n indicates the number of observations; β refers to the local regression parameters to be estimated; ε signifies a vector of independent and identically distributed error terms; ρ symbolizes the spatial autoregressive coefficient, measuring the intensity of dependence between cities. If no correlation is present, then ρ=0.

(2) Spatial error model (SEM)—The autocorrelation effect is considered to be a part of the residual structure. Assuming a spatial autocorrelation between error terms $\varepsilon_{i}$, the model form is as follows:

(6) Y=βX+ε, ε=λWε+ξ

where λ indicates the spatial autoregressive coefficient with the same meaning as ρ in Equation (5), and ξ represents the remaining part of the residual structure with no correlation.

When selecting a model, OLS was used to estimate the constrained model, without considering the influence of spatial correlation. The model selection is based on the significance of the Lagrange multiplier (LM). In accordance with the criterion proposed by Anselin [45], if LM(lag) and robust LM(lag) are more significant than LM(error) and robust LM(error), respectively, then the SLM is suitable; otherwise, SEM is preferred. The GeoDa software was used for the statistical processing and analysis of the data.

### 3.4. Model Implementation

Firstly, a zero-mean normalization method was applied to standardize all of the variables. Then, the Join Features tool in ArcGIS was used to transfer the attributes of the variables to the city spatial data. Before running GeoDa, Pearson’s correlation was calculated to analyze the degree of multi-collinearity problems for the explanatory variables. Multi-collinearity between the variables was defined as a value of 0.85 or higher. The results indicated that no pair of variables met the criteria for multi-collinearity. As a result, all of the variables were kept for further analysis. In addition, robust LM was applied to select the spatial regression model. Notably, GeoDa was implemented to qualify the association between the air quality and independent variables for eight economic zones.

## 4. Results and Discussion

### 4.1. Urban Growth Pattern Evaluation

**Figure 4:**
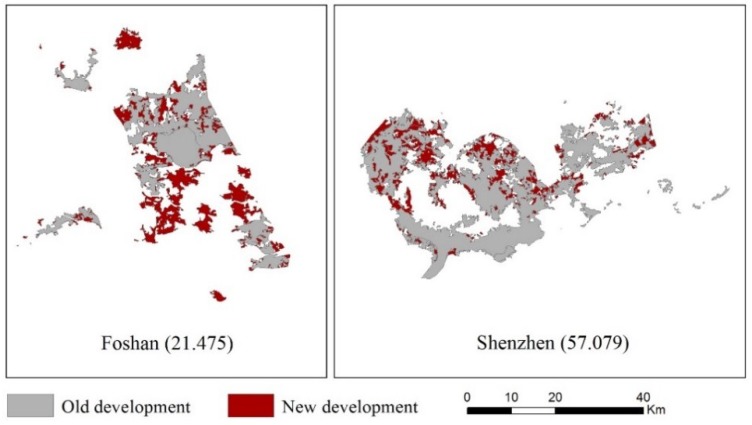
Examples of cities with low and high aggregated urban growth pattern index (AUGPI) value.

The total number of newly created patches in 2005–2015 reached 122,498, and the total area measured 3.326 $\times 10^{4}$ ${\mathrm{km}}^{2}$. The degree of urban aggregation at the city level was calculated through the AUGPI; a high value represents a high degree of urban aggregation and ceteris paribus. Figure 4 shows examples of the cities with low and high degrees of AUGPI. The cities with small values are mainly centralized in NWC, MRYLR, and NEC, indicating a comparatively severe urban sprawl in the region (Figure 2B).

### 4.2. Relationship between Urban Growth Pattern and Air Quality

In accordance with the robust LM results, SLM is more suitable than SEM for all of the economic zones, except for SCC. Therefore, SEM was implemented to qualify the relationship of SCC and SLM for the other seven zones, separately. Table 5 presents the regression results on the urban growth pattern and air quality.

The results show that urban growth pattern exerts a significant influence on air quality in NEC and NCC. The interpretation is that a more aggregated city will feature more exceedance days. To explain the results, two main potential reasons are discussed. Firstly, in the northern heating areas, a heating mode with coal as the main energy source significantly contributes to air pollution. Using Beijing as an example, heating has contributed a more than 50% increase in the concentration of PM_2.5_ in the winter months, since 2010 [46]. A city with high AUGPI value leads to a clumped population distribution because of the relatively short distance between destinations. A high demand for heating supply was observed in densely populated districts, leading to an additional coal consumption, which in turn affects the local air quality. Secondly, a high degree of urban aggregation results in heavy traffic congestion in NEC and NCC. In accordance with the traffic analysis report of major Chinese cities in 2016, announced by Mapabc, which is a widely recognized Chinese web mapping, navigation, and location-based service provider, 5 of the 10 most congested cities (i.e., Beijing, Changchun, Shenyang, Qingdao, and Dalian) are located in the zones. The AUGPI value of the five cities totaled 37.95, 50.40, 26.38, 34.40, and 41.62, respectively, which are all higher than the regional average level. Traffic congestion is related to the rapidly deteriorating urban air quality [17,18]. Hence, an increase in the degree of urban aggregation is significantly associated with poor air quality in NEC and NCC.

An opposite result was observed in SCC. The result indicates that aggregated cities are positively related with improved air quality. The result supports the compact city theory. In connection with the current situation in SCC, the potential explanations are summarized as follows: Through an emissions-based mechanism, SCC is highly developed in public transportation with a total length of 810 km urban rail transit lines by 2015, accounting for a quarter of the overall length in China, which provides support for public transit. Urban aggregation development enables an urban functional mixture of employment, recreation, and residence within proximity, features a high level of accessibility, and hence shortens the daily travel distance [47]. As a result, aggregated cities can reduce the fuel consumption for traffic and improve air quality by a decrease in the distance traveled and an increase in public transportation usage. On the basis of the data released by the China Forestry Database, the urban forest coverage for 2013 of the three provinces in SCC is comparatively high, with Fujian, Hainan, and Guangdong at 65.95%, 55.38%, and 51.26%, ranking first, fifth, and sixth of the 31 inter provinces, respectively. Less urban construction occupation has occurred in an aggregated city and a large area of green fields and has been recognized as highly related to improving the air quality reserve. Explanations from the two aspects may account for the significant and positive association between AUGPI and air quality in SCC.

Table 6 shows that urban diffusion is associated with improved air quality in NWC. Thus, the newly created areas of these cities constantly expand alongside valleys, because of terrain restrictions. For example, Lanzhou City, restricted by valley landform, extends similar to a strip along the river and is a typical linear city [48]. Air pollutants easily congregate and also stay for long periods in these cities. In general, although a scattered urban layout occupies additional open space, importantly, it creates wind paths, because of its low-density development, with which pollutants can be comparatively easily dispersed. Therefore, for these cities, a low value of urban aggregation is associated with good air quality.

Nonsignificant relationships were observed between the urban growth pattern and air quality in SCC, MRYLR, MRYTR, and SWC. These four zones feature one common characteristic, that is, they possess large populations. On the one hand, aggregated city development has worked efficiently on reducing private car dependence and vehicle miles traveled. On the other hand, a high degree of urban aggregation implies a massive usage of urban land and a concentration of human activities, resulting in an additional energy demand and consumption, which may offset the positive influence on air quality. The canceling effect may explain the nonsignificant relationship.

### 4.3. Relationship between Controlling Variables and Air Quality

The analysis shows that, in addition to the urban growth pattern, controlling the variables plays an important role on air quality. Table 7 presents the regression results.

From the results of land use mix, significant and negative relationships are found in NCC and MRYLR, significant and positive relationships in NWC, but nonsignificant relationships in the other five zones. Thus, the associations vary across the regions, and for most cities, land use mix causes no significant effect on air quality. Previous studies failed to find a significant association between the mixture and air quality, when considering the research area as a whole instead of delineating cities into different groups [14,23]. Recently, extensive attention has been paid to mixed use development in Chinese cities, to address severe problems caused by urban sprawl [49]. Notably, mixed land use is not a panacea, and the negative and nonsignificant effect on air quality should not be overlooked.

Compactness is negatively related to the number of exceedance days in NEC and SWC, indicating that compact urban form is associated with good air quality, which is expected to a certain extent. In this study, compactness reflects the regularity of the external form of the city, and high roundness indicates a compact city and limited travel distance. With the rapid expansion, city structures are complicated and fragmented. The degree of urban compactness may be impossible to measure comprehensively, accurately, and quantitatively by using a single index. Therefore, significant relationships between compactness and air quality are not observed for most cities. Future studies can adopt other indicators, such as the Boyce–Clark shape index, dual axis Fourier shape analysis, and fractal index, to measure the urban shape compactness at a comprehensive level, and to gain further in-depth information on the effect of urban shape on air quality.

The relationship between population density and air quality is under debate with two opposing views. Research conducted by Stone, R. [34], presented empirical evidence that an increase in density is associated with the reduction of air pollution on the basis of a study of 45 large U.S. metropolitan regions. However, other researchers have concluded that a large population density development led to high population-weighted PM_2.5_ concentrations on the basis of cross-sectional observations of 111 U.S. urban areas [24]. In this study, population density showed a positive and significant effect on air pollution in NCC and ECC, providing support for the second viewpoint. The potential explanation is that the cities in the two zones are associated with denser population distribution than other cities. The excessive concentration of population overburdens traffic load in urban areas, leading to heavy traffic congestion, which in turn contributes to additional vehicle exhaust emission.

Nonsignificant relationships were observed for the street connectivity and air quality for the eight economic zones, except for NCC. Contrary to the previously mentioned hypothesis, a good street connectivity is associated with a large road capacity and less traffic jam, resulting in less air pollutants. This nonsignificant relationship may be attributed to the indicator used in this article. Considering the availability of data, per capita urban road area was applied to represent the street connectivity, which fails to reflect the actual level of urban road development. A wide road is welcomed by the government and urban planners in China, leading to a significantly lower road length density than that of developed countries, maintaining the total road area. Further studies using the road length density index (unit: km/${\mathrm{km}}^{2}$) are needed to measure street connectivity.

The coefficient of the city size was expected to be positive, and results were consistent with this expectation. Since the implementation of the Reform and Opening-up policy, China has been experiencing a fast-paced development over the last four decades, with rapid urban land expansion as one of the main features. The direct consequence is the heavy loss of vegetation, which can reduce $O_{3}$ concentration, lower air temperatures, and remove air pollutants [19]. Therefore, a larger city size denotes that more open space will be occupied, and the air quality will worsen.

The regression results show that per capita GDP causes no significant effect on air quality for the eight zones. A high per capita GDP indicates a developed economy. On the one hand, in a wealthy city with a high per capita GDP, economic activities consume additional energy and increase the concentrations of harmful air pollutants. On the other hand, additional money has been devoted to the use of clean energy and the implementation of strict emission management to improve air quality in these cities. The contrasting relationship possibly causes a canceling effect. Thus, on average, per capita GDP causes a nonsignificant influence on air quality.

## 5. Conclusions

This study is a pioneering attempt to apply a spatial regression model by considering spatial autocorrelation to evaluate the relationship between air quality and the urban growth pattern in China, by conducting empirical research on 338 prefecture-level and above cities. To obtain local and accurate results, the conception of eight economic zones was adopted to delineate cities into different groups and to estimate regression separately. In addition, six urban form and socioeconomic indicators were applied as controlling variables. The results agree with the hypothesis that the urban growth pattern is associated with air quality. The findings are summarized as follows.

Firstly, the total number of newly created patches in 2005–2015 reached 122,498, whereas the total area measured 3.326 $\times 10^{4}$ ${\mathrm{km}}^{2}$. The AUGPI values ranged from 1.360 to 57.079 with a mean value of 24.939 and a median value of 25.771. The cities with small values are mainly centralized in NWC, MRYLR, and NEC, indicating a comparatively severe urban sprawl in the region.

Secondly, significant and positive relationships between AUGPI and air pollution were observed in NEC, NCC, and NWC, indicating that a high degree of urban aggregation is associated with poor air quality, whereas a negative parameter is obtained in SWC, showing an opposite association between urban aggregation and air quality. Nonsignificant connections were observed in the other four zones.

Thirdly, in terms of controlling the variables, significant and negative relationships between city size and air quality were found in half of the eight zones, indicating that a large city size is associated with poor air quality in Chinese cities. Population density is significantly correlated with poor air quality in NCC and ECC. The associations between land use mix and air quality vary across regions, and for most cities, land use mix causes no significant effect on air quality. Nonsignificant associations between per capita GDP and air quality were derived for all of the zones, because of the canceling effect. Compactness and connectivity were found to be nonsignificant with air quality for most cities, because of data restriction.

Nowadays, air pollution is a crucial problem in China and has become an inevitable threat to human health. The findings significantly highlighted that urban growth pattern, land use mix, population density, and city size exert important but different influences on air quality across the eight economic zones. China is still undergoing rapid urbanization, and an improved understanding of the quantitative relationships between urban forms and air quality is important for urban planners to formulate efficient strategies at the planning stage for the government to create alternative policies to improve air quality. Finally, considering the availability of data, only a summary metric (AQI) was used to reflect the air quality. AQI failed to reveal the relationships between individual air pollutants and urban forms. When further detailed air quality data become available, future research can be conducted to address this issue.

## Figures and Tables

**Table 1:** Main urban form variables identified in literature review.

Category	Variables
Landscape	Number of urban patches
	Mean urban patch area
	Total urban area
	Largest patch index
	Standard deviation of urban patches
	Eccentricity standard deviation ellipse
	Total forest area
	Forest mixing
	Fractal dimension index
	Boyce–Clark shape index
	Shape compactness
	Landscape shape index
	Contiguity
	Patch cohesion index
	Mean perimeter area ratio
Population	Degree of population centering
	Total population amount
	Population density
Mixture	Land use mix
Accessibility	Street connectivity

Summarized from [14,23,24,29,30,31].

**Table 2:** Air quality index (AQI) categories (HJ 633-2012).

AQI Value	Air Pollution Level	Impacts on Health
0–50	Good	Pollution poses little or no risk.
51–100	Moderate	The air quality is acceptable; certain pollutants exert a weak effect on sensitive groups.
101–150	Slightly polluted	Situation becomes worse for sensitive groups; healthy groups begin to feel uncomfortable.
151–200	Moderately polluted	The air is dangerous for the heart and respiratory system.
201–300	Heavily Polluted	Everyone may begin to experience health problems.
301–500	Severely Polluted	The air pollution phenomenon severely threatens public health.

**Table 3:** Variable description and data resources. AUGPI—aggregated urban growth pattern index.

Variables	Description	Data Resource	Data Year
Exceedance days	AQI > 100	Chinese Ministry of Ecology and Environment	2015
AUGPI	Aggregated urban-growth pattern index	National Land Use/Cover Database of China	2005–2015
Mixed value	Land use mix	Baidu Maps	2015
Compactness	Urban shape compactness	National Land Use/Cover Database of China	2015
Density	Population density (per ${\mathrm{km}}^{2}$)	China Urban Construction Statistical Yearbook	2015
Connectivity	Street connectivity (%)	China Urban Construction Statistical Yearbook	2015
City size	City size (${\mathrm{km}}^{2}$)	National Land Use/Cover Database of China	2015
Per capita GDP	Per capita GDP (yuan)	China Urban Construction Statistical Yearbook	2015

**Table 4:** Descriptive statistics for the variables.

Variables	Min	Max	Mean	Std. Dev
Exceedance days	0.000	295.000	82.152	62.452
AUGPI	1.360	57.079	24.939	10.190
Mixed value	0.271	2.264	2.030	0.163
Compactness	0.032	0.488	0.111	0.066
Density	49.734	10,711.667	3012.452	2256.826
Connectivity	0.001	0.248	0.058	0.045
City size	15.500	12,187.000	472.434	980.424
Per capita GDP	10,601.000	195,792.000	59,150.088	32,806.974

**Table 5:** Descriptive statistics for eight economic zones. ECC—eastern coastal China; MRYLR—middle reaches of the Yellow River; MRYTR—middle reaches of the Yangtze River; NCC—northern coastal China; NEC—Northeast China; NWC—Northwest China; SCC—southern coastal China; SWC—Southwest China.

Zone Division	Included Provinces	$\mathrm{GDP}\;(10^{8}\;\mathrm{yuan})$	$\mathrm{Population}\;(10^{4})$	Mean Exceedance Days
ECC	Jiangsu, Shanghai, and Zhejiang	118,332.4	15,852	100.71
MRYLR	Henan, Inner Mongolia, Shanxi, and Shaanxi	77,636	19,305	123.69
MRYTR	Anhui, Hubei, Hunan, and Jiangxi	82,548	23,042	77.35
NCC	Beijing, Hebei, Shandong, and Tianjin	116,857	20,653	179.71
NEC	Heilongjiang, Jilin, and Liaoning	54,442	10,976	83.53
NWC	Gansu, Ningxia, Qinghai, Tibet, and Xinjiang	20,102	6930	70.67
SCC	Fujian, Guangdong, and Hainan	87,070	15,313	22.11
SWC	Guangxi, Guizhou, Sichuan, Yunan, and Chongqing	73,023	23,985	40.63

**Table 6:** Relationship between urban growth pattern and air quality.

Zone Division	Coefficient	Std. Error	T-Statistic	Probability
NEC	0.145	0.076	1.913	0.056 *
NCC	0.038	0.175	−1.862	0.063 *
SCC	−0.054	0.032	−1.666	0.095 *
NWC	0.413	0.155	2.670	0.007 **
ECC	0.089	0.059	1.493	0.135
MRYLR	0.124	0.095	1.315	0.189
MRYTR	−0.019	0.069	−0.271	0.786
SWC	0.028	0.050	0.562	0.574

* Significant at the *p* < 0.10 level. ** Significant at the *p* < 0.05 level.

**Table 7:** Relationship between controlling variables and air quality.

Zone Division	Mixed Value	Compactness	Density	Connectivity	City Size	Per capita GDP
NEC	−0.186	−0.152 **	0.076	−0.116	0.173 *	0.070
NCC	−0.647 **	−0.185	0.838 **	−0.978 *	0.055	−0.094
SCC	0.038	−0.037	−0.003	0.047	0.299 **	−0.064
NWC	0.132 *	0.053	0.001	0.210	0.261	−0.138
ECC	−0.218	0.036	0.463 **	−0.141	−0.011	0.102
MRYLR	−0.259 *	0.064	0.099	0.069	0.898 *	−0.083
MRYTR	0.0841	−0.240	0.092	0.097	0.350 *	0.127
SWC	0.161	−0.142 **	0.116	−0.089	−0.009	0.103

* Significant at the *p* < 0.10 level. ** Significant at the *p* < 0.05 level.
